# Chasing Relief, Finding Pain: A Case of Medication Overuse Headache

**DOI:** 10.1192/j.eurpsy.2026.11090

**Published:** 2026-07-31

**Authors:** T. Grahovac Juretić, A. Mančić, E. Žderić, K. Ružić, E. Dadić-Hero

**Affiliations:** Department of psychiatry, University Hospital Centre Rijeka, Rijeka, Croatia

## Abstract

**Introduction:**

Medication overuse headache (MOH), also known as rebound headache, is a chronic headache disorder resulting from the frequent and excessive use of analgesics intended for acute pain or migraine management. One such preparation, frequently prescribed when standard pain relievers are insufficient, is a compound analgesic consisting of paracetamol, propyphenazone, caffeine, and codeine phosphate. While effective for short-term treatment of moderate acute pain, prolonged or excessive use may lead to dependency and worsening of headache symptoms. The codeine component, a morphine derivative, carries a particular risk of addiction, as approximately 10% of codeine is metabolized into morphine by hepatic enzymes.

**Objectives:**

The aim of this case report is to describe the development of dependence on a codeine-containing compound analgesic in a patient with pre-existing psychiatric comorbidities and to examine the clinical consequences of medication overuse headache in such a context.

**Methods:**

We present the case of a 62-year-old female patient with a ten-year history of psychiatric treatment for anxiety and depressive symptoms. Data were collected from clinical interviews, medical records, and psychiatric evaluations. Her pharmacological treatment included duloxetine 60 mg daily and pregabalin 150 mg nightly. Despite this regimen, the patient reported daily use of 3–6 tablets of the compound analgesic for migraine, cervical, and spinal pain.

**Results:**

The patient developed persistent headaches that did not improve despite escalating intake of the compound analgesic. Instead, her anxiety and agitation intensified in parallel with her reliance on the medication. This clinical picture is consistent with MOH, complicated by codeine misuse. Genetic variability in hepatic enzyme activity may have influenced the metabolism of codeine to morphine, possibly explaining the lack of analgesic efficacy despite increasing doses. Consequently, the patient experienced no meaningful pain relief, reinforcing the cycle of overuse and psychological distress.

**Conclusions:**

This case illustrates how psychiatric comorbidities can interact with analgesic misuse to perpetuate MOH and codeine dependence. Clinicians should remain vigilant for signs of overuse, particularly in patients with underlying mental health disorders. Could genetic variability in codeine metabolism explain the ineffectiveness of escalating doses in certain patients, and should this inform future personalized approaches to pain management?

**Disclosure of Interest:**

None Declared

